# Dual-functional xanthan gum/chitosan nanocomposite doped with copper oxide nanoparticles for high-capacity adsorption of methylene blue and methyl orange dyes and antimicrobial applications

**DOI:** 10.1039/d6ra02416e

**Published:** 2026-08-12

**Authors:** Ahmed K. Saleh, Hussain Alenezi, Jehan S. Albrahim, Feng F. Hong

**Affiliations:** a State Key Laboratory of Advanced Fiber Materials, College of Biological Science and Medical Engineering, Donghua University No. 2999 North Renmin Road Shanghai 201620 China; b Scientific Research Base for Bacterial Nanofiber Manufacturing and Composite Technology, China Textile Engineering Society Shanghai 201620 China; c Cellulose and Paper Department, National Research Centre 33 El-Behouth St., Dokki, P.O. 12622 Giza Egypt asrk_saleh@yahoo.com; d Manufacturing Engineering Technology Department, College of Technological Studies, PAAET Shuwaikh 70654 Kuwait; e Department of Biology, College of Science, Princess Nourah bint Abdulrahman University P. O. Box 84428 Riyadh 11671 Saudi Arabia

## Abstract

In the current study, a xanthan gum/chitosan/copper oxide nanoparticle (XaG/Ch/CuO-NPs) nanocomposite was synthesized and evaluated as a biosorbent for organic dyes and antimicrobial activity. The resulting XaG/Ch/CuO-NPs nanocomposite was characterized to confirm the successful incorporation and uniform dispersion of CuO-NPs within the nanocomposite. The removal efficiencies of methylene blue (MB) and methyl orange (MO) were investigated as a function of pH, contact time, and dye concentration. Under the optimized conditions, the XaG/Ch/CuO-NPs nanocomposite exhibited high adsorption capacities of 88 and 114 mg g^−1^ for MB and MO, respectively. The close agreement between experimental data and the pseudo-second-order kinetic model (*R*^2^ = 0.99) suggests that chemical interactions govern the adsorption process. The adsorption equilibrium followed the Langmuir model, suggesting monolayer adsorption on a homogeneous surface. The adsorption capacity retention rate reaches 85% after 5 cycles. The antimicrobial activities of the XaG/Ch and XaG/Ch/CuO-NPs nanocomposite were evaluated against *Escherichia coli*, *Salmonella typhimurium*, *Staphylococcus aureus*, *Streptococcus mutans*, and *Candida albicans*. The findings showed that the XaG/Ch/CuO-NPs nanocomposite exhibited broad-spectrum inhibitory effects against all tested pathogens, with inhibition zones ranging from 15 to 12 mm, whereas the XaG/Ch composite displayed no detectable antimicrobial activity. Overall, the XaG/Ch/CuO-NPs nanocomposite is promising for industrial wastewater purification, offering a sustainable alternative to conventional adsorbents.

## Abstract 2

In this study, a xanthan gum/chitosan/copper oxide nanoparticle (XaG/Ch/CuO-NPs) nanocomposite was synthesized and investigated as a biosorbent for organic dyes and an antimicrobial agent.

## Graphical abstract

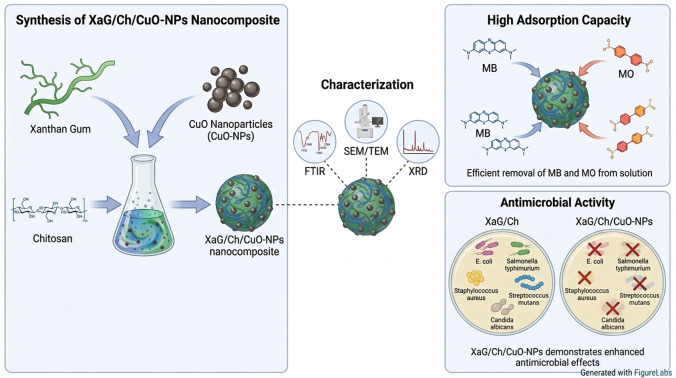

## 1. Introduction

The rapid expansion of industrial activities, including textiles, cosmetics, leather, pharmaceuticals, and food processing, has led to the release of large quantities of organic dyes into water bodies.^1^ These dyes pose serious environmental threats due to their complex molecular structures, high stability, and the toxic by-products formed during partial degradation. Consequently, the removal of organic dyes from industrial effluents has become a major challenge for researchers and environmental scientists to maintain cleaner water.^1^

Adsorption is widely regarded as an effective treatment method because of its simplicity, low cost, and high efficiency. Recent research has focused on replacing conventional commercial adsorbents with more sustainable and economical materials derived from natural biomass.^2^ Biomass-based materials such as cellulose,^3^ starch,^4^ lignin,^5^ alginate,^6^ and Ch^7^ have attracted attention as effective dye adsorbents because of their abundance, biocompatibility, biodegradability, hydrophilicity, low production costs, and rich chemical functionality. Their structural diversity and physicochemical properties make them ideal candidates for interacting with complex dye molecules while minimizing secondary pollution.

Hydrogels and three-dimensional porous structures fabricated from natural polymers, including tree gums, offer improved robustness and durability, which are advantageous for water remediation. The occurrence of multiple active sites in these polymeric structures facilitates dye adsorption and the formation of stable complexes in aqueous systems. Hydrogels derived from biopolymers such as Ch, alginate, pectin, carrageenan, XaG, and chondroitin sulfate have been widely used due to their high swelling capacity and tunable surface chemistry.^8^ XaG, when combined with Ch, forms a stable polyelectrolyte hydrogel that overcomes the limitations of each polymer.^9^ Ch, a biodegradable amino polysaccharide obtained from chitin, is valued for its low cost, ease of modification, and favorable stability; its charge density varies with pH due to protonation or deprotonation of amino groups. XaG, rich in carboxyl groups, complements Ch by providing additional functional sites and structural enhancement.^10^ XaG–Ch hydrogel exhibits pH-responsive behavior: at low pH, protonated Ch imparts a positive charge, while at high pH, deprotonated XaG introduces a negative charge. These charge variations, along with hydrogen bonding, significantly improve the mechanical and chemical stability of the hydrogel and support its reusability without compromising performance.^11^

CuO-NPs are among the most important transition metal oxides in the rapidly advancing field of nanotechnology. Their growing relevance is attributed to their versatile properties and broad applications. CuO-NPs can be prepared using a range of synthesis routes, including green and conventional chemical methods, each offering distinct advantages in simplicity, environmental sustainability, and economic feasibility.^12^ In green synthesis of CuO-NPs, plant extracts and microbial supernatant filtrates serve as eco-friendly, cost-effective reducing and stabilizing agents. These biological materials facilitate nanoparticle formation without the need for toxic chemicals, making the process both sustainable and efficient.^13^ Chemical methods for the synthesis of CuO-NPs typically rely on reducing agents such as ascorbic acid or glucose to convert copper salts into CuO-NPs.^14^ CuO-NPs exhibit a broad spectrum of biological applications, including antimicrobial, cytotoxic, and antiviral activities, as well as anti-inflammatory, antioxidant, and wound-healing activities.^15,16^ In contrast, CuO-NPs are widely employed in various industrial applications, including dye removal, heavy metal remediation, and photocatalytic wastewater treatment.^17,18^ Additionally, they are utilized in sensors, solar cells, batteries, and catalytic processes due to their excellent chemical stability and high surface reactivity.^19^ Given the multifunctional properties of CuO-NPs, many researchers have incorporated them for the decoration and functionalization of various composite materials. For instance, Saleh and his colleagues developed a bacterial cellulose/Ch/PVA nanocomposite incorporating CuO-NPs. These hybrid nanocomposites demonstrated strong antimicrobial activity and exhibited notable *in vitro* anticancer effects against colon, hepatocellular, and breast cancer cell lines. Remarkably, the CuO-NP enhanced nanocomposites also showed potent larvicidal activity, causing mortality across all larval instars and the pupal stage. All these diverse biological activities are related to CuO-NPs.^20^ Another study demonstrated the fabrication of PVA/polyethylene oxide/CuO-NPs nanocomposites using the solution-casting technique. The incorporation of CuO-NPs markedly enhanced the dielectric constant, AC conductivity, and dielectric loss of the films compared with the pristine PVA/polyethylene oxide blend, with these properties further increasing as the CuO-NP content was raised.^21^ A PVA/polyvinyl pyrrolidone/CuO-NP composite nanomaterial was synthesized *via* a precipitation method and subsequently applied for malachite green uptake. The composite nanomaterials exhibited a maximum adsorption capacity of 76.92 mg g^−1^, highlighting their strong potential for dye removal applications.^22^

Therefore, the present study aimed to fabricate a novel XaG/Ch nanocomposite functionalized with CuO-NPs, yielding XaG/Ch/CuO-NPs nanocomposites. A range of analytical techniques, including SEM, EDX, TEM, XRD, and FT-IR, were employed to verify the synthesis and structural characteristics of the developed nanocomposites. For industrial applications, the XaG/Ch/CuO-NPs nanocomposite was evaluated for its efficiency in removing both anionic and cationic dyes. In the biomedical context, the nanocomposite was further assessed for its antimicrobial potential against a variety of pathogenic microorganisms, highlighting its multifunctional applicability.

## 2. Experimental

### 2.1. Materials

Xanthan gum (XG), chitosan (medium molecular weight, degree of deacetylation >75%), and copper(ii) sulfate pentahydrate were obtained from Sigma-Aldrich. All other analytical-grade chemicals were procured from commercial suppliers and used without further purification.

### 2.2. Preparation of XaG/Ch and XaG/Ch/CuO-NPs nanocomposite

**Fig. 1:**
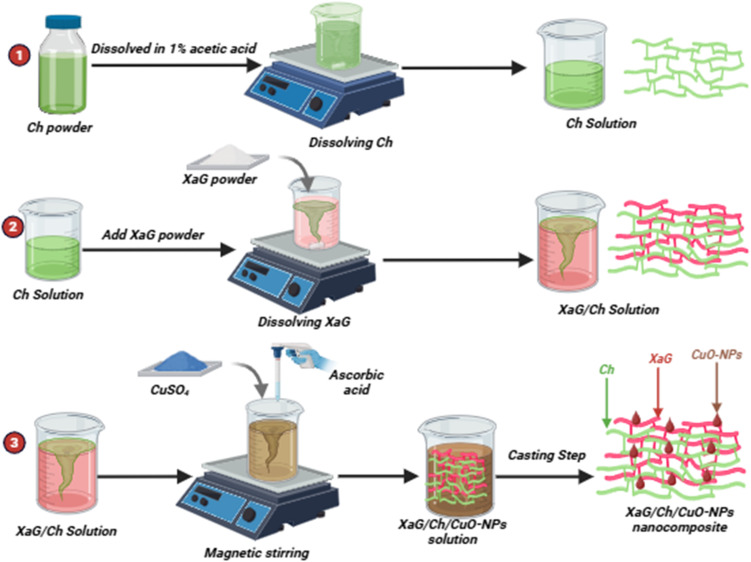
Schematic illustration depicting the fabrication process of XG/Ch and XaG/Ch/CuO-NPs nanocomposites.

The preparation of XaG/Ch and XaG/Ch/CuO-NPs nanocomposites were carried out according to the procedure of Tabassum *et al.*, with minor changes^23^ as illustrated in Fig. 1. In brief, Ch powder was dissolved in (1% w/v) in 1% (v/v) aqueous acetic acid with continuous stirring at room temperature until a homogeneous solution was obtained. After complete dissolution of Ch, XaG powder was gently added under continuous stirring, and the mixture was stirred for 30 min to confirm complete hydration of XaG. An aqueous solution of CuSO_4_·5H_2_O (0.1 M) was therefore added dropwise to the homogenized XaG/Ch mixture under permanent stirring. To facilitate the *in situ* reduction of Cu^2+^ ions to CuO-NPs, a freshly prepared ascorbic acid solution (0.05 M) was added gradually under constant stirring. The reduction was indicated by a gradual change in the color of the dispersion, confirming nanoparticle formation. The nanocomposites were fabricated through a solution casting method.

### 2.3. Characterization

#### 2.3.1. Fourier-transform infrared spectrometer (FT-IR)

The chemical interactions and functional groups of the XaG/Ch and XaG/Ch/CuO-NPs nanocomposites were characterized by FT-IR spectroscopy using a Shimadzu 8400 s instrument (Japan) equipped with IR Solution software for spectral analysis. Measurements were carried out at room temperature in the range of 4000–500 cm^−1^, with a spectral resolution of 4 cm^−1^, and each spectrum was recorded as the average of 16 scans using KBr pellet preparation.

#### 2.3.2. X-ray diffraction (XRD)

X-ray diffraction analysis was performed using a Rigaku diffractometer equipped with Cu Kα radiation (*λ* = 1.5406 Å) and operated at 40 kV and 30 mA to evaluate the crystalline structure and phase composition of the prepared hybrid material. The diffraction patterns were recorded over a 2*θ* range of 5–80° at a scanning rate of 2° min^−1^. The crystallite size of CuO nanoparticles was calculated from the main diffraction peaks using the Scherrer equation, *D* = *Kλ*/*β* cos *θ*, where *D* is the crystallite size, *K* is the shape factor (0.9), *λ* is the X-ray wavelength, *β* is the full width at half maximum in radians, and *θ* is the Bragg angle.

#### 2.3.3. Transmission electron microscope (TEM)

Ultra High-Resolution TEM (JEOL-JEM 2010, Japan) was used to examine the XaG/Ch/CuO-NPs nanocomposites for investigating the formation, shape, and size of CuO-NPs.

#### 2.3.4. Scanning electron microscopy (SEM)

The surface morphology and particle dispersion of the nanocomposites were analyzed by SEM (JEOL JSM-IT200) at an accelerating voltage of 15 kV. Prior to imaging, the samples were gold-sputtered using a K500X coater (UK) to improve surface conductivity, while elemental composition and the occurrence of copper were checked through Energy Dispersive X-ray Spectroscopy (EDS).

#### 2.3.5. Thermogravimetric analysis (TGA)

The thermal stability and degradation behavior of the prepared nanocomposites were evaluated using a thermal analyzer under a nitrogen flow, with the samples heated from room temperature to 600 °C at a constant rate of 10 °C min^−1^.

### 2.4. Adsorption studies of MB and MO dyes

A comprehensive set of batch adsorption experiments was performed in triplicate to assess the adsorption behavior of MB and MO onto XaG/Ch/CuO-NPs nanocomposites. The influence of key operational parameters, including initial dye concentration, contact time, solution pH, and temperature, was systematically investigated. Stock solutions of MB and MO (1000 mg L^−1^) were prepared using deionized water and subsequently diluted to obtain working solutions with concentrations ranging from 50 to 600 mg L^−1^. All adsorption experiments were conducted in borosilicate glass vials under continuous magnetic stirring at 500 rpm to ensure uniform dispersion of the adsorbent. The pH of the dye solutions was adjusted to the desired values using 0.1 M HCl or 0.1 M NaOH prior to the adsorption process. A predetermined mass of the XaG/Ch/CuO-NPs nanocomposites was added to a known volume of dye solution and agitated for the specified contact time. Upon reaching adsorption equilibrium, the mixtures were centrifuged to separate the solid phase, and the residual concentrations of MB and MO in the supernatant were determined by UV-visible spectrophotometry at their respective maximum adsorption wavelengths (*λ*_max_ = 664 nm for MB and 464 nm for MO). The equilibrium adsorption capacity (*q*_e_, mg g^−1^) was calculated using eqn (1):1
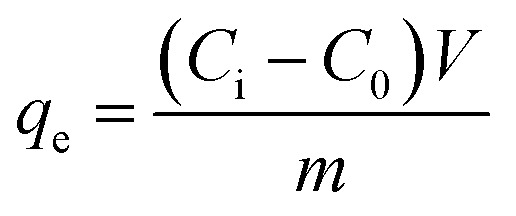
where *C*_i_ and *C*_0_ (mg L^−1^) represent the initial and equilibrium dye concentrations, respectively, *V* (L) denotes the volume of the dye solution, and *m* (g) refers to the mass of the XaG/Ch/CuO-NPs nanocomposite employed. All adsorption experiments were independently repeated three times under identical conditions, and the results are reported as mean values ± standard deviation. The relative standard deviation was also calculated to evaluate experimental reproducibility, confirming good consistency of the measurements. Error bars representing standard deviation have been included in the relevant figures.

### 2.5. Antimicrobial activity

#### 2.5.1. Microbial strains

The prepared XaG/Ch and XaG/Ch/CuO-NPs nanocomposites were investigated for antimicrobial properties against five pathogens. All microbes were purchased from the American Type Culture Collection (ATCC). The organisms tested were *Staphylococcus aureus* (*S. aureus*) ATCC 25923 and *Streptococcus mutans* (*S. mutans*) ATCC 25175, as Gram-positive, while *Escherichia coli* (*E. coli*) ATCC 25922 and *Salmonella typhimurium* (*S. typhimurium*) ATCC 14028, as Gram-negative bacteria, and *Candida albicans* (*C. albicans*) ATCC 10231, representing the unicellular fungi.

#### 2.5.2. Preinoculum preparation

A purified single colony from the tested microbes was inoculated into a 100 mL Erlenmeyer flask containing 25 mL sterilized nutrient broth (prepared by dissolving 13 g in 1000 mL of distilled water) and then incubated at 37 °C under shaking at 200 rpm for 24 h for bacteria and 48 h for unicellular fungi.

#### 2.5.3. Disc diffusion technique

The antimicrobial activities of the XaG/Ch and XaG/Ch/CuO-NPs nanocomposites were assessed using the disc diffusion method following the protocol of Saleh *et al.*, with minor modifications.^24^ Initially, about 15 mL of sterilized nutrient agar (prepared by dissolving 23 g in 1000 mL of distilled water) was poured into Petri dishes and left to solidify. A microbial suspension (10^8^ CFU per mL), adjusted to the density of a 0.5 McFarland standard, was then evenly spread over the agar surface, followed by placing 10 mm discs of the XaG/Ch and XaG/Ch/CuO-NPs nanocomposites onto the inoculated plates. The inoculated plates were first incubated at 4 °C for 2 hours to allow adequate diffusion of the test samples and to suppress microbial activity temporarily. They were then incubated at 37 °C for 24 hours for bacterial strains and 48 hours for the unicellular fungus. Following incubation, antimicrobial activity was assessed by measuring the diameters of the inhibition zones, including the nanocomposite disc. All results represent the mean values of triplicate experiments and are expressed in millimeters (mm). Prior to use, all nanocomposite discs were sterilized under UV light for 30 min to maintain aseptic conditions.

### 2.6. Statistical analysis

All quantitative results are presented as mean ± standard deviation (SD). Statistical analysis was performed using one-way ANOVA followed by Tukey's post hoc test in GraphPad Prism 9.0. A *p*-value less than 0.05 was considered statistically significant.

## 3. Results and discussions

### 3.1. Characterization of nanocomposites

#### 3.1.1. FT-IR spectra

**Fig. 2:**
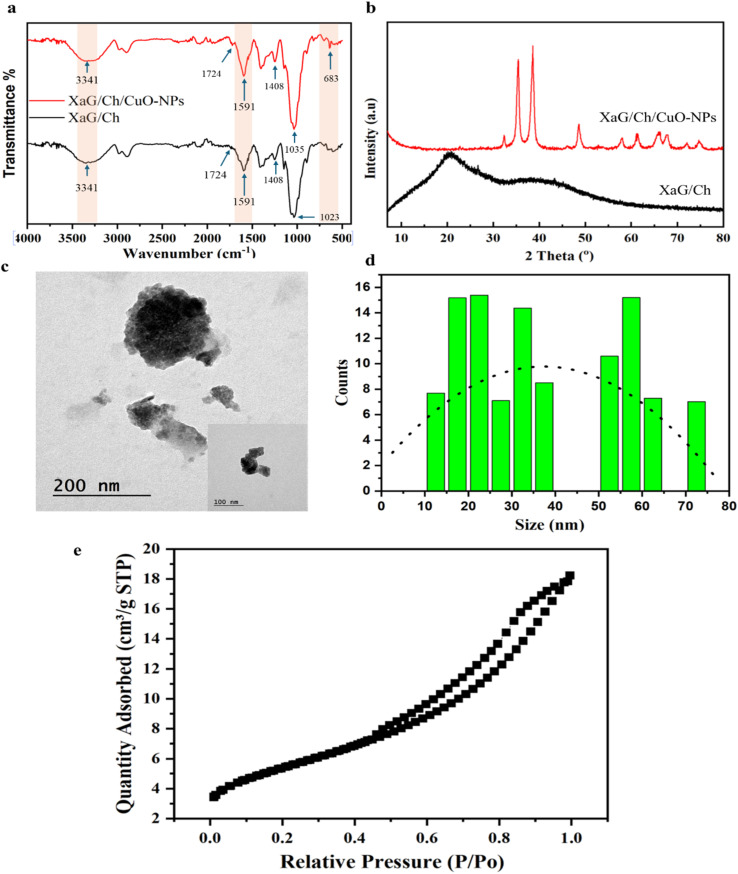
Structural and morphological characterization of XaG/Ch and XaG/Ch/CuO-NPs nanocomposites: (a) FTIR spectra (b) XRD (c) TEM image of CuO-NPs, (d) particle-size distribution obtained from TEM analysis, and (e) N_2_ adsorption/desorption isotherm.

The FT-IR spectra of the XaG/Ch blend and the XaG/Ch/CuO-NPs nanocomposite (Fig. 2a) showed characteristic bands of the polysaccharide skeleton and distinct modifications induced by copper nanoparticle incorporation. XaG/Ch blend exhibited broad band at 3341 cm^−1^, which corresponds to O–H and N–H stretching vibrations, demonstrating extensive hydrogen bonding between Ch chains and XaG.^25,26^ Moreover, the peak at 1724 cm^−1^ is assigned to C=O stretching of residual acetyl or carboxyl groups, while the band at 1591 cm^−1^ represents N–H bending of Ch –NH_2_ groups.^27^ Moreover, the position of this group reflects the electrostatic interaction between the protonated –NH_3_^+^ groups of Ch and the –COO^−^ groups of XG, verifying polyelectrolyte complex development.^23^ In addition, the peak at 1408 cm^−1^ corresponds to symmetric COO^−^ stretching, and the band at 1023 cm^−1^ arises from C–O–C and pyranose ring vibrations of the polysaccharide backbone. After Cu nanoparticle incorporation, evident spectral changes appeared in the XaG/Ch/CuO-NPs nanocomposites spectrum, including broadening of the 3341 cm^−1^ band, which indicates intensified hydrogen bonding and possible coordination between Cu and –OH/–NH_2_ groups. The amine-associated band shifts from 1591 cm^−1^ to 1590 cm^−1^, and changes in the 1408 cm^−1^ band suggest interactions between Cu and carboxylate groups. The polysaccharide band shifts from 1023 cm^−1^ in XaG/Ch to 1035 cm^−1^ in XaG/Ch/CuO-NPs nanocomposites, indicating changes in the structural environment following Cu incorporation. Importantly, a distinct new band appears at 683 cm^−1^, corresponding to Cu–O or Cu–NP lattice vibrations, which confirms the formation of stable copper nanoparticles within the hydrogel matrix. Collectively, these changes demonstrate that Cu nanoparticles are well incorporated through coordination and physical entrapment without disrupting the native polymer backbone.^28^

#### 3.1.2. XRD analysis

The XRD patterns of XaG/Ch and XaG/Ch/CuO-NPs nanocomposites are shown in Fig. 2b. The XaG/Ch sample exhibited a broad diffraction halo centered at approximately 2*θ* = 20–22°, indicating the predominantly amorphous/semi-crystalline nature of the XaG/Ch polymeric matrix. This broad reflection is commonly associated with the limited ordering of polysaccharide chains and the intermolecular interactions among the –OH, –NH_2_, and –COOH groups of xanthan gum and chitosan (DOI: https://doi.org/10.1016/j.ijbiomac.2019.06.243). On the other hand, the XRD pattern of the XaG/Ch/CuO-NPs nanocomposite displayed several sharp diffraction peaks, confirming the successful formation of crystalline CuO nanoparticles within the biopolymer matrix. The main diffraction peaks observed at approximately 2*θ* = 32.5°, 35.5°, 38.7°, 48.7°, 53.5°, 58.3°, 61.5°, 66.2°, 68.1°, 72.4°, and 75.1° can be assigned to the monoclinic CuO crystalline phase. These reflections correspond mainly to the (110), (−111), (111), (−202), (020), (202), (−113), (−311), (113), (311), and (400) crystallographic planes, respectively. The obtained diffraction pattern confirms the monoclinic tenorite structure of CuO nanoparticles, in agreement with JCPDS card No. 96-901-5569.^29^ The presense of the characteristic CuO diffraction peaks with the broad polymeric background confirms that CuO-NPs were successfully generated and incorporated into the XaG/Ch network without disrupting the main polymer structure. Moreover, no additional intense impurity peaks were observed, indicating that CuO was the dominant crystalline copper oxide phase in the nanocomposite. The crystallite size of CuO-NPs was estimated from the main indexed XRD peaks using the Scherrer equation. The calculated crystallite sizes ranged from 10.80 to 26.23 nm, with an average value of approximately 15.58 nm, confirming the nanoscale crystalline nature of CuO nanoparticles within the XaG/Ch matrix.

#### 3.1.3. TEM analysis

The synthesized CuO NPs were characterized using TEM (Fig. 2c). At low magnification, the CuO-NPs appear as dense aggregates, indicating a high degree of agglomeration caused by their high surface energy and strong interparticle attractions, as is typical for metal oxide nanoparticles obtained *via* chemical reduction. At higher magnification, individual particles may be seen with shapes that are almost spherical or slightly elongated. Their uniform contrast and well-defined edges show that they are crystalline and that nucleation worked well during synthesis.^30,31^ The particle-size distribution obtained from TEM image analysis sing ImageJ software is shown in Fig. 2d. The CuO-NPs exhibited a broad size distribution in the range of 10–75 nm, with most particles located between 15 and 60 nm. This broad distribution is consistent with the agglomerated morphology observed in the TEM image. The presence of smaller primary nanoparticles together with larger particle domains indicates that the prepared CuO-NPs tend to form secondary aggregates. This behavior is commonly attributed to the high surface energy of CuO nanoparticles and the absence of strong steric stabilization during drying.

#### 3.1.4. Surface area

The N_2_ adsorption/desorption isotherm of the prepared XaG/Ch/CuO-NPs sample is shown in Fig. 2e. The isotherm exhibited a gradual increase in nitrogen uptake with increasing relative pressure, reaching approximately 18 cm^3^ g^−1^ STP at P/P_0_ close to 1.0. The presence of a clear hysteresis loop at medium to high relative pressure indicates mesoporous behavior, which is commonly associated with capillary condensation within mesopores and interparticle voids. Therefore, the observed isotherm supports the formation of a porous/agglomerated CuO-containing structure, where interparticle voids may provide accessible pathways for dye adsorption and interaction with active –OH, –NH_2_, –COO^−^, and CuO surface sites.

#### 3.1.5. SEM analysis

**Fig. 3:**
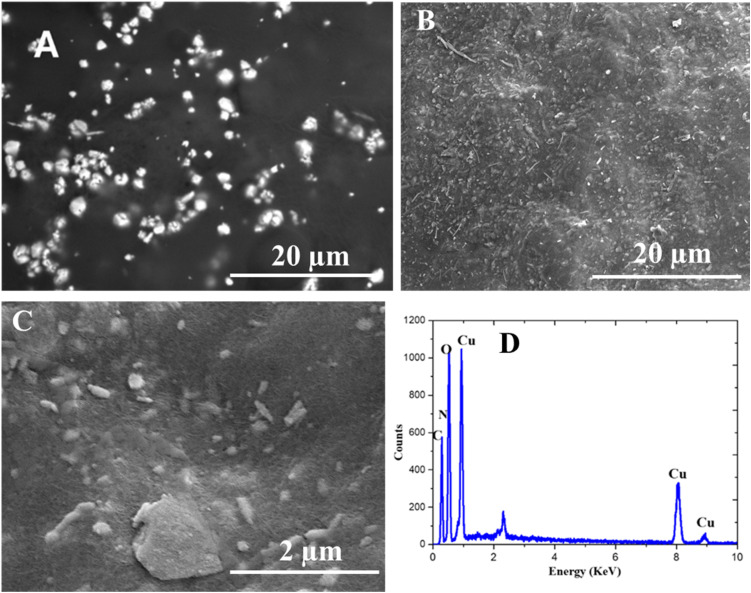
SEM micrograph of (A) XaG/Ch, (B and C) XaG/Ch/CuO-NPs nanocomposites with different magnification, and (D) EDX spectrum of XaG/Ch/CuO-NPs nanocomposites.

Fig. 3 presents SEM micrographs of the XaG/Ch blend and the XaG/Ch/CuO-NPs nanocomposites at different magnifications, along with the corresponding EDX spectrum confirming elemental composition. The microstructure of the XaG/Ch hydrogel (Fig. 3A) exhibits a moderately smooth and homogeneous surface morphology, typical of well-integrated biopolymeric networks formed through electrostatic interactions between Ch and XaG. Only minor irregularities and dispersed micro-domains are visible, indicating good miscibility between Ch and XaG for stable polyelectrolyte complex formation. In contrast, the incorporation of copper nanoparticles markedly alters the surface morphology. At low magnification (Fig. 3B), the XaG/Ch/CuO-NPs nanocomposites exhibit a noticeably rougher and more heterogeneous texture, with bright, granular features distributed across the matrix. These bright spots correspond to copper nanoparticles embedded within the polymeric framework.^32^ The increased surface roughness suggests enhanced structural reinforcement and potential changes in cross-linking density attributable to Cu–polymer interactions. A higher-magnification image (Fig. 3C) further highlights the presence of well-dispersed copper nanoparticles throughout the composite matrix. The CuO-NPs appear as distinct agglomerated and non-agglomerated bright clusters, indicating both micro- and nanoscale distribution. Their stable embedding within the hydrogel network confirms success *in situ* reduction and nucleation of CuO-NPs. The EDX spectrum of XaG/Ch/CuO-NPs nanocomposites (Fig. 3D) provides elemental confirmation of copper incorporation. Strong Cu peaks appear prominently, accompanied by carbon and oxygen peaks originating from the polysaccharide backbone. Quantitative EDX analysis confirmed the successful incorporation of CuO nanoparticles into the XaG/Ch matrix, with Cu accounting for 63.46 wt% (27.33 atomic%) of the total elemental composition. The presence of Cu signals verifies that metallic nanoparticles were successfully integrated within the XaG/Ch matrix during synthesis.^23^

#### 3.1.6. TGA analysis

**Fig. 4:**
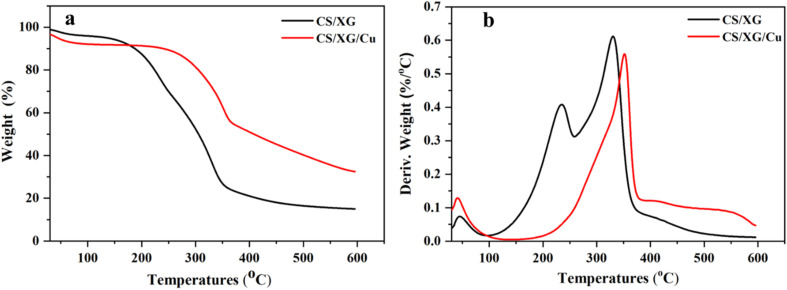
TGA (a) DTG (b) curves for XaG/Ch and XaG/Ch/CuO-NPs nanocomposites.

The thermal stability and degradation behavior of the XaG/Ch and XaG/Ch/CuO-NPs nanocomposites were evaluated using TGA (Fig. 4a) and DTG (Fig. 4b). Both samples exhibited multistage decomposition, typical of polysaccharide-based hydrogel. In the first stage (below 120 °C), a small weight loss was observed for both samples, corresponding to the evaporation of physically adsorbed and bound water. The XaG/Ch/CuO-NPs nanocomposites showed slightly lower weight loss in this region than XaG/Ch, indicating a more compact network and reduced moisture retention due to the presence of CuO-NPs, which may enhance intermolecular interactions. The second major decomposition stage occurred between 200 and 350 °C, associated with the degradation of the Ch and XaG backbones. For the XaG/Ch blend, this stage was pronounced, with significant mass loss beginning around 220 °C and peaking sharply at approximately 300 °C, as shown in the DTG curve. This event corresponds to the cleavage of glycosidic linkages, depolymerization, and decomposition of side groups (*e.g.*, acetyl, pyranose units). The XaG/Ch/CuO-NPs nanocomposites, however, exhibited a more gradual mass loss and a slightly shifted DTG maximum (around 320–330 °C), indicating that CuO-NPs improved thermal stability. This enhancement may be attributed to coordination interactions between Cu and functional groups (–OH, –NH_2_, –COOH), which restrict polymer mobility and delay chain scission. The third degradation stage observed between 350–450 °C represents the breakdown of more thermally resistant fragments and carbonaceous residues. For XaG/Ch/CuO-NPs nanocomposites, the DTG peak in this region appeared narrower and occurred at a higher temperature compared to the XaG/Ch blend, further confirming improved structural integrity in the presence of CuO-NPs. The reduced intensity of the DTG peak also suggests that Cu may promote char formation, which acts as a protective barrier and slows thermal decomposition. Above 450–600 °C, both materials exhibited slow, continuous mass loss associated with the decomposition of residual carbonaceous content. Notably, the XaG/Ch/CuO-NPs nanocomposites retained a higher final mass (32%) than the XaG/Ch hydrogel, probable due to the presence of thermally stable inorganic Cu/CuO residues.^23^

### 3.2. Adsorption of cationic and anionic dyes

The adsorption behavior of both cationic and anionic dyes was systematically investigated using the newly developed XaG/Ch/CuO-NPs nanocomposites. This bio-based adsorbent exhibited remarkable efficiency in removing representative organic dyes from aqueous media. To elucidate the adsorption mechanisms, a series of batch experiments was conducted under varying experimental conditions, including pH, adsorption time, initial dye concentration, and temperature. These investigations provided valuable insights into the surface chemistry and interaction pathways governing dye uptake by the composite material.

#### 3.2.1. Effect of pH

**Fig. 5:**
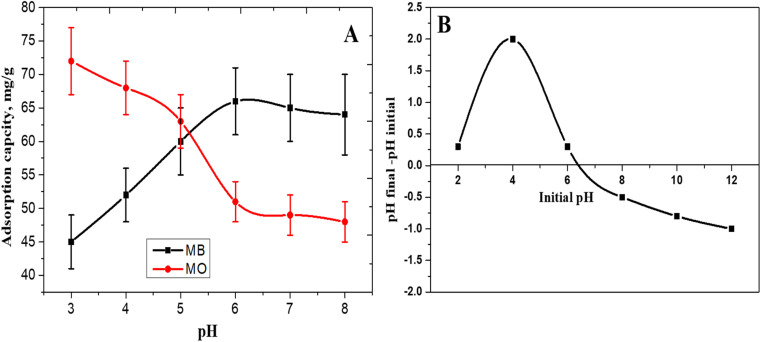
Effect of pH and adsorbent dose of XaG/Ch/CuO-NPs nanocomposites on MB and MO adsorption (A) and point zero charge of XaG/Ch/CuO-NPs nanocomposites (B).

Solution pH is widely recognized as a key factor influencing adsorption processes, primarily because it alters both the surface charge of the adsorbent and the ionization state of the dye molecules. As shown in Fig. 5, the adsorption capacities of MB and MO onto the XaG/Ch/CuO-NPs nanocomposites exhibited strong pH dependence. For MB, the uptake increased from 45 mg g^−1^ at pH 3 to a maximum of approximately 66 mg g^−1^ at pH 6, followed by a slight decline at higher pH values. In contrast, the adsorption of MO displayed the opposite trend, with the highest adsorption capacity (72 mg g^−1^) achieved at pH 3, which gradually decreased to around 48 mg g^−1^ as the pH increased to 8. This distinct pH-responsive behavior can be explained by the ionization of functional groups within the polymeric matrix and the ionic characteristics of the dye molecules. Ch contains primary amino groups that become protonated (–NH_3_^+^) under acidic conditions, rendering the surface positively charged. Consequently, electrostatic repulsion occurs between the protonated Ch surface and cationic MB molecules, thereby reducing adsorption. Conversely, strong electrostatic attraction arises between these positively charged –NH_3_^+^ groups and the negatively charged sulfonate (–SO_3_^−^) groups of MO, enhancing its adsorption at low pH. As the pH increases above 5–6, deprotonation of Ch amino groups and ionization of XaG carboxylic groups occur, forming negatively charged sites (–COO^−^). This transformation enhances the electrostatic attraction toward cationic MB molecules while simultaneously diminishing the affinity for anionic MO due to charge repulsion. In addition to electrostatic forces, MB adsorption is further stabilized through hydrogen bonding and coordination interactions between its aromatic rings and the hydroxyl and amino functionalities of the biopolymer matrix.^33^ The pH-dependent adsorption profiles confirm the amphoteric and multifunctional nature of the XaG/Ch/CuO-NPs nanocomposites, which enables efficient removal of both cationic and anionic dyes through complementary electrostatic and specific interaction mechanisms. These results are consistent with previous findings on karaya gum/Ch sponges, which demonstrated efficient adsorption performance toward both MO and MB.^11^

The point of zero charge (pH_p_zc) is an important parameter that provides insight into the surface charge characteristics of the XaG/Ch/CuO-NPs nanocomposites and its interaction with charged pollutants. Based on the chemical composition of the nanocomposit, the pH_p_zc was predicted to be approximately 6.4, as shown in Fig. 1B. This value reflects the balance between the carboxylic groups (–COOH) of xanthan gum, which tend to impart a negative surface charge, and the amino groups (–NH_2_) of chitosan together with the hydroxylated surface sites of CuO nanoparticles, which contribute positive surface charges under acidic conditions.

At pH values lower than 6.4, the amino groups of chitosan become protonated to form –NH_3_^+^ species, resulting in a positively charged surface. In contrast, when the solution pH exceeds 6.4, progressive deprotonation of carboxylic and surface hydroxyl groups occurs, generating negatively charged sites. Consequently, the composite surface becomes increasingly negative, which promotes the adsorption of cationic pollutants through electrostatic interactions. The predicted pH_p_zc value of 6.4 suggests that the xanthan gum/chitosan/CuO composite possesses amphoteric surface properties and can effectively interact with both positively and negatively charged molecules depending on the solution pH.

#### 3.2.2. Effect of contact time

**Fig. 6:**
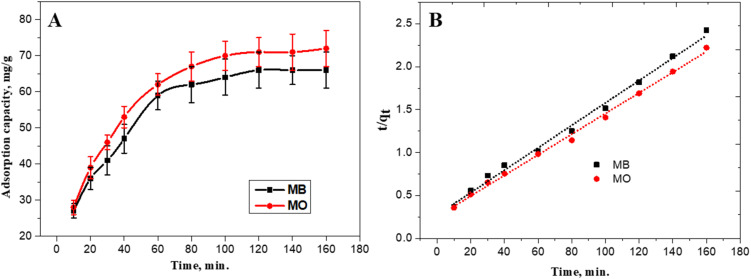
Relation between time and adsorption capacity of MB and MO (A) and Pseudo second-order kinetics matching of the adsorption process (B) by XaG/Ch/CuO-NPs nanocomposites.

Evaluating the adsorption capacity of the XaG/Ch/CuO-NPs nanocomposites as a function of contact time is essential to understand the adsorption kinetics and equilibrium behavior.^34^Fig. 6A illustrates the variation in adsorption efficiency with contact time for both MB and MO, along with the corresponding kinetic model fittings. The adsorption capacity of the nanocomposite increased progressively with time for both dyes, indicating a rapid initial uptake followed by a gradual approach to equilibrium. A noticeable plateau was observed after approximately 80 min, beyond which no further significant increase in dye removal was observed, suggesting that adsorption sites had become saturated. The equilibrium adsorption capacities were determined to be 62 mg g^−1^ for MB and 67 mg g^−1^ for MO, respectively.

The high adsorption efficiency of the XaG/Ch/CuO-NPs nanocomposites can be attributed to their unique structural and chemical characteristics. The composite exhibits a rough, porous morphology with many accessible active sites arising from abundant functional groups, including carboxylate (–COO^−^), hydroxyl (–OH), and amino (–NH_2_) moieties. The negatively charged carboxylate groups promote strong electrostatic attraction with the cationic MB molecules, thereby enhancing dye uptake. In addition, MB can form hydrogen bonds with hydroxyl and amino groups within the biopolymer matrix, further stabilizing its adsorption. For the anionic dye MO, the positively charged amino groups (–NH_3_^+^) within the Ch serve as active binding sites, enabling electrostatic attraction with the sulfonate groups of MO molecules.

Furthermore, hydrogen-bonding interactions between dye molecules and the functional groups of XaG and Ch (hydroxyl, carboxyl, and amino groups) also contribute to adsorption.^35^ The time-dependent adsorption behavior confirms that the XaG/Ch/CuO-NPs nanocomposites exhibit a multifunctional adsorption mechanism, where both electrostatic and hydrogen-bonding interactions synergistically govern the uptake of cationic and anionic dyes.^36^ The relatively short equilibrium time (80 min) indicates the high accessibility and reactivity of the surface functional sites, further emphasizing the potential of this composite as an efficient and rapid biosorbent for organic dye removal.

The experimental data of adsorption mechanism onto XaG/Ch/CuO-NPs nanocomposites were studied utilizing the most popular kinetic models: the pseudo-first-order and pseudo-second-order models and intra-particle mass transfer diffusion model (eqn (2)–(4)), respectively). These models are expressed as follows:2
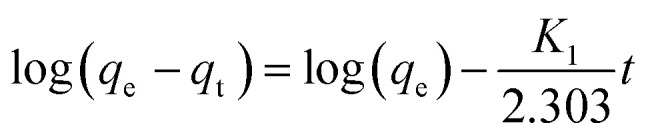
3
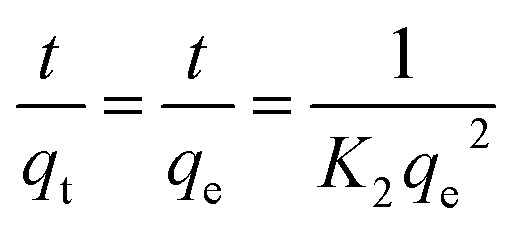
4
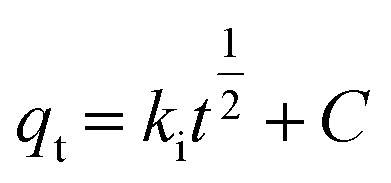
In these models, *q*_e_ and *q*_t_ (mg g^−1^) correspond to the amounts of dye adsorbed at equilibrium and at a given contact time *t* (min), respectively, while *k*_1_ (min^−1^) and *k*_2_ (g mg^−1^ min^−1^) represent the rate constants of the pseudo-first-order and pseudo-second-order kinetic models. Also, ki is the intra-particle diffusion rate constant (mg g^−1^ min^0.5^) and C (mg g^−1^) is the intercept which can be calculated from the slope of the linear plots of *q*_t_*versus*
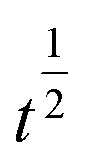
. The adsorption kinetics of MB and MO onto the XaG/Ch/CuO-NPs nanocomposites were analyzed by fitting the experimental data to the three kinetic models. The resulting linear fittings are presented in Fig. 6B, and the derived kinetic parameters are listed in Table 1.

**Table 1:** Kinetic factors for dyes adsorption by XaG/Ch/CuO-NPs nanocomposites

Models	Parameters	MB	MO
Pseudo first order	*q*_e,cal_ (mg g^−1^)	61.9	58.9
	*K*_1_ (min^−1^)	0.005	0.007
	*R* ^2^	0.92	0.93
Intra particle diffusion model	C (mg g^−1^)	18.7	20.3
	*K*_i_ (mg g^−1^ min^−1 0.5^)	4.3	4.6
	*R* ^2^	0.89	90

The pseudo-second-order model provided a significantly better representation of the experimental data, yielding high correlation coefficients (*R*^2^ ≈ 0.99) for both dyes, whereas the pseudo-first-order model and intra-particle mass transfer diffusion model exhibited comparatively lower correlation values (*R*^2^ = 0.92 and 89 for MB and 0.93 and 90 for MO, respectively). In addition, the equilibrium adsorption capacities predicted by the pseudo-second-order model closely matched the experimentally determined values, further supporting its applicability. The superior performance of this model suggests that the adsorption process is predominantly controlled by chemisorption mechanisms, involving electron-sharing or electron-transfer interactions between the dye molecules and the functional groups present on the XaG/Ch/CuO-NPs nanocomposite surface. Consequently, the rate-controlling step is associated with chemical interactions such as surface complexation, electrostatic attraction, and hydrogen bonding with active functional sites (–NH_2_, –COOH, and –OH) within the biopolymeric matrix.^36^ The kinetic study demonstrates that the adsorption of both cationic and anionic dyes follows a pseudo-second-order mechanism, implying that chemisorption dominates the overall dye removal process on the XaG/Ch/CuO-NPs nanocomposite surface.

#### 3.2.3. Effect of dye concentration and equilibrium isotherm

**Fig. 7:**
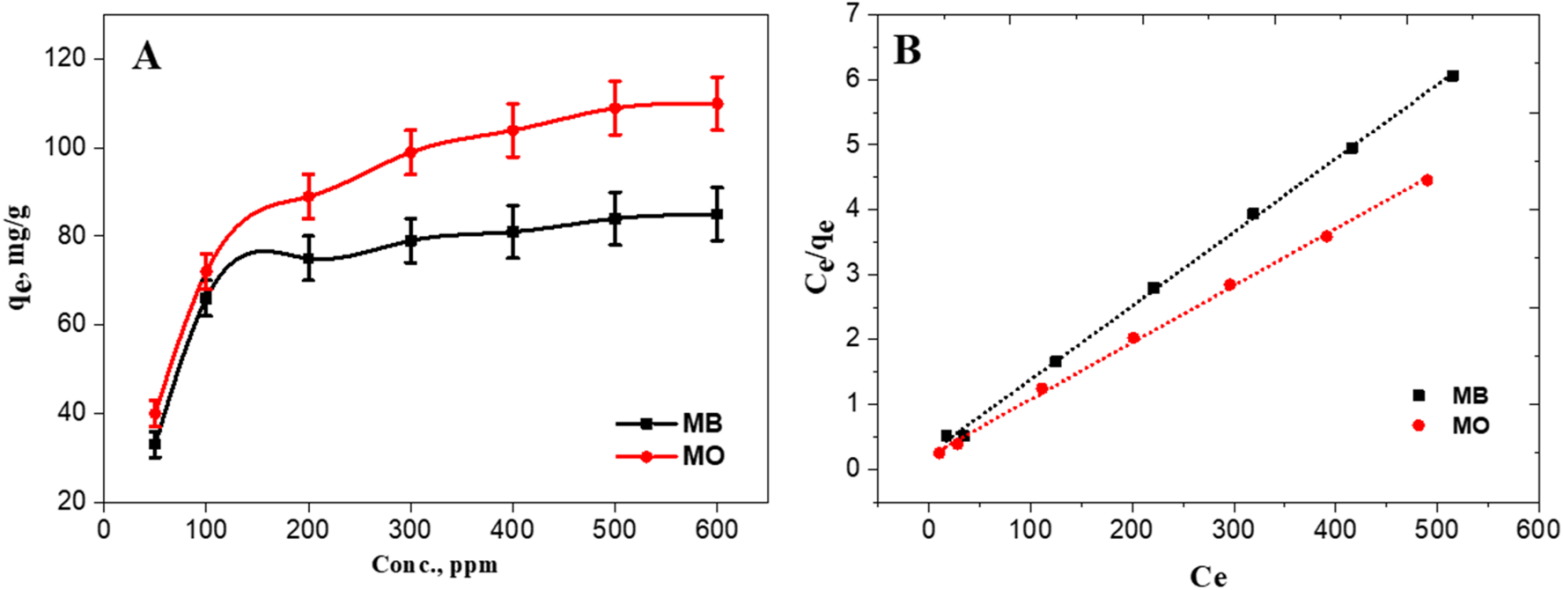
(A) The relationship between initial dye concentration and adsorption efficiency of MB and MO onto XaG/Ch/CuO-NPs nanocomposites, and (B) Langmuir adsorption isotherm fitting.

The adsorption performance of the XaG/Ch/CuO-NPs nanocomposites was examined under different initial dye concentrations to explore how concentration affects the adsorption behavior.^37^ As illustrated in Fig. 7A, the adsorption capacity for both MB and MO increased significantly with higher dye concentrations. This pattern results from the stronger mass-transfer driving force at elevated concentrations, which promotes the movement of dye molecules toward the active adsorption sites on the nanocomposite surface. At an initial dye concentration of 500 mg L^−1^, the maximum adsorption capacities reached 84 mg g^−1^ for MB and 109 mg g^−1^ for MO, indicating the strong affinity and effective adsorption ability of the synthesized material. The adsorption isotherms of the XaG/Ch/CuO-NPs nanocomposites were analyzed using both the Langmuir and the Freundlich models.

To describe the equilibrium adsorption data, the Langmuir and the Freundlich isotherm models were applied. The Langmuir model assumes monolayer adsorption on a homogeneous surface with a finite number of identical sites (eqn (5)).^38^5
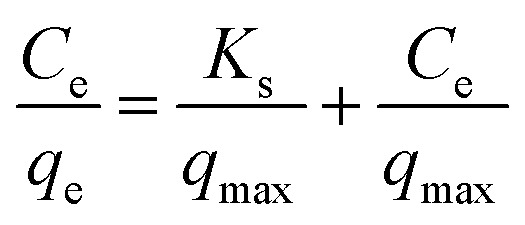
Here, *C*_e_ is the equilibrium dye concentration (mg L^−1^), *q*_e_ is the equilibrium adsorption capacity (mg g^−1^), *q*_max_ is the maximum monolayer capacity (mg g^−1^), and *K*_s_ represents the Langmuir affinity constant (L mg^−1^). The results obtained (Fig. 7B and Table 2) revealed that the experimental data for both dyes fitted well with the Langmuir model, as evidenced by the high correlation coefficients (*R*^2^ > 0.98). The by XaG/Ch/CuO-NPs nanocomposites exhibited remarkable maximum adsorption capacities of 88 mg g^−1^ for MB and 114 mg g^−1^ for MO, indicating a strong and uniform affinity of the functionalized surface toward both dyes. This excellent fit suggests a monolayer adsorption mechanism dominated by electrostatic and coordination interactions between the dyes and the active sites of the nanocomposite. The Freundlich isotherm model was also applied to examine the surface heterogeneity of the adsorbent, as expressed in eqn (6):6
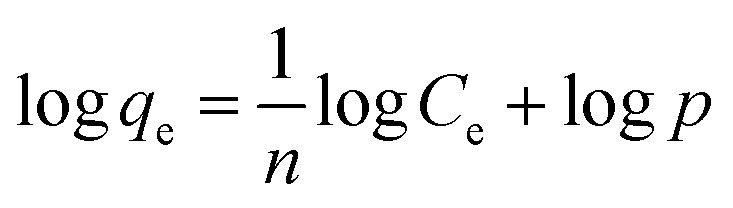
where *P* is the Freundlich constant indicative of adsorption capacity, and 1/*n*represents the heterogeneity factor. The calculated parameters (Table 2) showed *P* values of 22.9 and 26.9, and *n* values of 4.48 and 3.06 for MB and MO, respectively. However, the relatively lower *R*^2^ values (0.87 and 0.95) compared to those of the Langmuir model confirm that the adsorption process is better represented by Langmuir behavior rather than by the Freundlich model. These findings suggest that adsorption of both dyes onto the by XaG/Ch/CuO-NPs nanocomposites occurs predominantly through monolayer coverage on a homogeneous surface, while minor heterogeneity effects are also present due to the composites' multifunctional groups.^39^

**Table 2:** Calculated equilibrium parameters obtained from Langmuir and the Freundlich isotherm models for dyes adsorption onto XaG/Ch/CuO-NPs nanocomposites

Models	Parameters	MB	MO
Langmuir	*q*_m_ (mg g^−1^)	88	114
	*K*_s_ (L mg^−1^)	21.9	23.8
	*R* ^2^	0.99	0.99
Freundlich	*P* (mg g^−1^)	22.9	26.9
	*n*	4.48	3.06
	*R* ^2^	0.87	0.95

#### 3.2.4. Thermodynamics study

The thermodynamic characteristics of dye adsorption onto the XaG/Ch/CuO-NPs nanocomposites were investigated by performing batch adsorption experiments at three different temperatures (25, 35, and 45 °C) under optimal pH conditions. Each system was allowed to reach equilibrium over a contact time of 4 h, after which the equilibrium adsorption capacity was calculated using eqn (1). The influence of temperature on adsorption efficiency was analyzed to determine key thermodynamic parameters, including changes in enthalpy (Δ*H*°), entropy (Δ*S*°), and Gibbs free energy (Δ*G*°). The values of Δ*G*° were obtained using the Van't Hoff equation (eqn (8)), whereas Δ*H*° and Δ*S*° were estimated from the slope and intercept of the linear relationship between ln *K*_c_ and 1/*T*, respectively (eqn (9)).

7 Δ*G*° = −*RT* ln *K*_c_

8
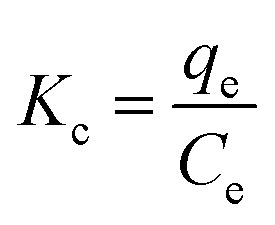
Here, *K*_c_ denotes the distribution coefficient constant, *R* is the universal gas constant (8.314 J mol^−1^ K^−1^), and *T* represents the absolute temperature in Kelvin.9
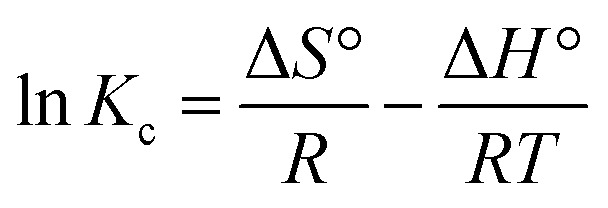
where Δ*H*° and Δ*S*° values were calculated from plotting the linear relation Ln *K*_c_*vs.*1/*T*.

**Fig. 8:**
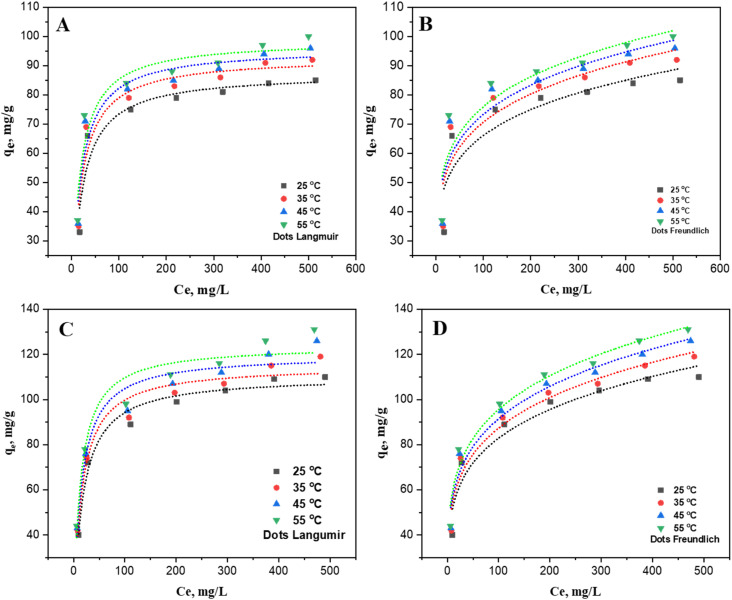
Adsorption isotherm curves of MB and MO on XaG/Ch/CuO-NPs nanocomposites at different temperatures. Experimental data and non-linear fitting using the Langmuir model (A and C) and the Freundlich model (B and D) are presented, respectively.

The results obtained demonstrated that the adsorption capacities of both dyes increased with rising temperature, representing an endothermic adsorption method. Fig. 8 exhibited that, at an initial dye concentration of 500 mg L^−1^, the adsorption performance increased from 85 mg g^−1^ to 96 mg g^−1^ for MB and from 109 mg g^−1^ to 126 mg g^−1^ for MO as the temperature rose from 25 °C to 45 °C. This enhancement in adsorption capacity with temperature may originate from the increased mobility and diffusion rate of dye molecules across the liquid–solid interface. Moreover, the reduced viscosity of the aqueous phase smooths the transport of dye molecules into the internal pores of the adsorbent. Also, activation of additional adsorption sites may be enhanced at elevated temperatures. The observed increase in adsorption capacity with increasing temperature clearly shows that the adsorption method is endothermic, a behavior commonly reported for dye adsorption on biopolymer-based and metal-functionalized adsorbents.^4^ The thermodynamic parameters (Table 3) showed negative Δ*G*° values, confirming that the adsorption of both MB and MO onto the XaG/Ch/CuO-NPs nanocomposites is spontaneous. Moreover, the positive Δ*H*° further supports the endothermic nature of the process, while the positive Δ*S*° shows an increase in randomness at the solid–liquid interface through dye adsorption. Overall, the thermodynamic findings reveal that dye adsorption onto XaG/Ch/CuO-NPs nanocomposites is a spontaneous, endothermic, and entropy-driven process, dominated by strong electrostatic and chemical interactions between dyes and composite functional groups.

**Table 3:** Calculated thermodynamic parameters (Δ*G*°, Δ*H*°, and Δ*S*°) describing the adsorption of MB and MO on XaG/Ch/CuO-NPs nanocomposite at various temperatures

Dyes	Δ*H*° (kJ mol^−1^)	Δ*S*° (kJ mol^−1^ K^−1^)	Δ*G*° (kJ mol^−1^)			
			298 K	308 K	318 K	328 K
MB	8.8	35.2	−1.6	−2	−2.4	−2.5
MO	8.7	37.2	−2.4	−2.7	−3	−3.14

**Fig. 9:**
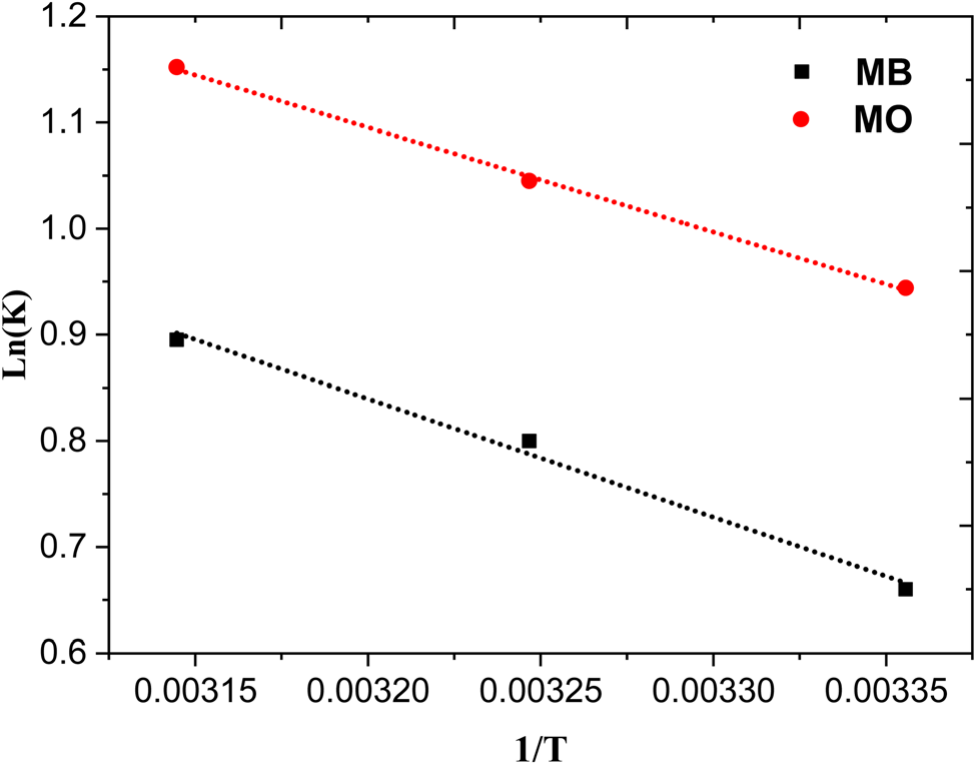
Van't Hoff plot of ln(*K*) *vs.* 1/*T* (A) and reusability of XaG/Ch/CuO-NPs nanocomposites for the adsorption of MB and MO (B).

The thermodynamic parameters were derived from Van't Hoff plots (Fig. 8A), and the calculated values are summarized in Table 3. For both MB and MO, the Gibbs free energy (Δ*G*°) values were negative at all tested temperatures, confirming that the adsorption processes are spontaneous. For MB adsorption, the Δ*G*° converted increasingly negative with rising temperature, indicating that higher temperatures enhance adsorption, consistent with an endothermic process. Similarly, MO adsorption also exhibited negative Δ*G*° values; however, their magnitudes were smaller than those of MB, suggesting a less spontaneous and thermodynamically weaker adsorption affinity. Similar trends have been reported for dye adsorption onto Ch-based hydrogels and metal oxide-functionalized biopolymers, where temperature promotes stronger dye adsorbent interactions. Similar trends have been reported for dye adsorption onto Ch-based material, where temperature promotes stronger dye–adsorbent interactions.^40^ The positive enthalpy change (Δ*H*°) observed for MB adsorption confirms that the process is endothermic, requiring energy input to proceed efficiently. The relatively large Δ*H*° value further implies that the adsorption is chemisorption, characterized by strong interactions such as electrostatic attraction or complexation between MB molecules and the functional groups on the nanocomposite surface (Fig. 9).

In contrast, the smaller Δ*H*° value for MO suggests a weaker endothermic process, typical of physisorption mechanisms dominated by van der Waals forces or hydrogen bonding. Furthermore, the positive entropy change (Δ*S*°) for MB adsorption indicates an increase in randomness at the solid–liquid interface during the adsorption process. This enhancement in entropy is attributed to the desolvation of MB molecules and the rearrangement of interfacial water structure upon adsorption. For MO, a smaller positive Δ*S*° value reflects limited disruption of interfacial water and relatively weaker interaction with the nanocomposite surface. Overall, these results confirm that the adsorption of both dyes onto XaG/Ch/CuO-NPs nanocomposite is spontaneous and thermodynamically favorable, with MB adsorption being more endothermic and chemically driven, while MO adsorption follows a predominantly physical mechanism.

The adsorption performance of the developed adsorbent for MO and MB removal was compared with that of various previously reported composite adsorbents, as summarized in Table 4. This comparison highlights the effectiveness, adsorption capacity, and practical applicability of the present material relative to existing systems reported in the literature.

Comparison of the adsorption efficiencies of XaG/Ch/CuO-NPs nanocomposites toward MO and MB with those reported for other adsorbent systems in the literature

**Table:** 

Adsorbent	Dye	Optimum conditions	Maximum adsorption	Reference
XaG/Ch/CuO-NPs	MO	pH 3, temperature 298 K, time 80 min, MO conc. 109 mg g^−1^	114 mg g^−1^	The current study
	MB	pH 6, temperature 298 K, time 80 min, MO conc. 84 mg g^−1^	88 mg g^−1^	
Ch/polyaniline/CuO-NPs	MO	Time 45 min, pH 4, at room temperature	94.6%	41
CNF/Silica-NH_2_/Ag-NPs	MB and MO	pH 6 for MB and 3 for MO, time 60 min, dye conc. 300 ppm, temperature 25 °C	MB (370 mg g^−1^) and MO (151 mg g^−1^)	42
Polypyrrole@magnetic- Ch	MO	pH 4.5, time 40 min, temperature 25 °C	95 mg g^−1^	43
TEMPO/CNF/Ch/Ag-NPs	MB	pH of 7 with a contact time of 60 min at 88 to 90 mg g^−1^ of MB	432 mg g^−1^	44
Poly(methyl methacrylate)/GO/CuO	MB and MO	pH 6.5, time 120 min, temperature 25 °C, dye Conc. 15 ppm	MB (84%) and MO (35%)	45

**Table:** 

Adsorbent	Other pollutants	Optimum conditions	Maximum adsorption	Reference
XaG/Ch	Malachite green	Box-Behnken design: Ch 1.75 mg L^−1^, XaG (1.73–3.0%), dye conc. (5–27.5 mg L^−1^), pH 6, time (5–7.5 min), and stirring rate (105 rpm)	99.7%	46
XaG/Ch/Au-NPs	Reduction of nitrobenzene	Solvent water, 10 mg of catalyst, time 60 min, and temperature 50 °C	96%	47
XaG/Ch	Cd^2+^, Ni^2+^, and Cu^2+^	Adsorbent 50 mg, pH 6, and metal concentration 50 mg L^−1^	Cd^2+^ (152.33 mg g^−1^), Ni^2+^ (144.79 mg g^−1^), and Cu^2+^ (139.71 mg g^−1^), respectively	48

### 3.3. Adsorption mechanism

**Fig. 10:**
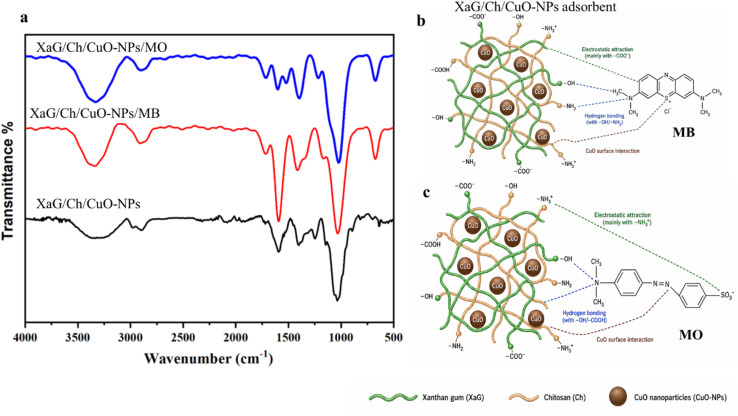
FTIR spectra and proposed adsorption mechanisms of MB and MO onto the XaG/Ch/CuO-NPs nanocomposite. (a) FTIR spectra of XaG/Ch/CuO-NPs before and after adsorption of MB and MO. (b) Proposed adsorption mechanism of cationic MB and (c) anionic MO onto XaG/Ch/CuO-NPs.

The FTIR spectra of XaG/Ch/CuO-NPs before and after adsorption are shown in Fig. 10a. Clear changes were observed after MB and MO adsorption, especially in the broad –OH/–NH stretching region around 3340 cm^−1^ and in the region near 1590, 1408, and 1035 cm^−1^. These changes indicate the participation of hydroxyl, amino, carboxylate, and polysaccharide backbone groups in dye binding. The variation in the intensity and position of these bands after adsorption suggests that the dye molecules interact with the XaG/Ch/CuO-NPs surface through electrostatic attraction, hydrogen bonding, and possible interactions with CuO surface sites. The proposed adsorption mechanisms are illustrated in Fig. 10b and c. For MB, a cationic dye, adsorption is mainly promoted by electrostatic attraction between positively charged MB molecules and negatively charged –COO^−^ groups of xanthan gum, especially when the adsorbent surface becomes more negatively charged. In addition, hydrogen bonding between MB and –OH/–NH_2_ groups of the XaG/Ch matrix may further stabilize the adsorbed molecules, while CuO nanoparticles can provide additional surface interaction sites. For MO, an anionic dye, adsorption is mainly favored by electrostatic attraction between the negatively charged sulfonate group of MO and protonated NH_3_^+^ groups of chitosan under acidic conditions. Hydrogen bonding with –OH/–COOH groups and possible CuO surface interactions may also contribute to MO uptake.^49,50^

### 3.4. Antimicrobial activity of nanocomposites

**Fig. 11:**
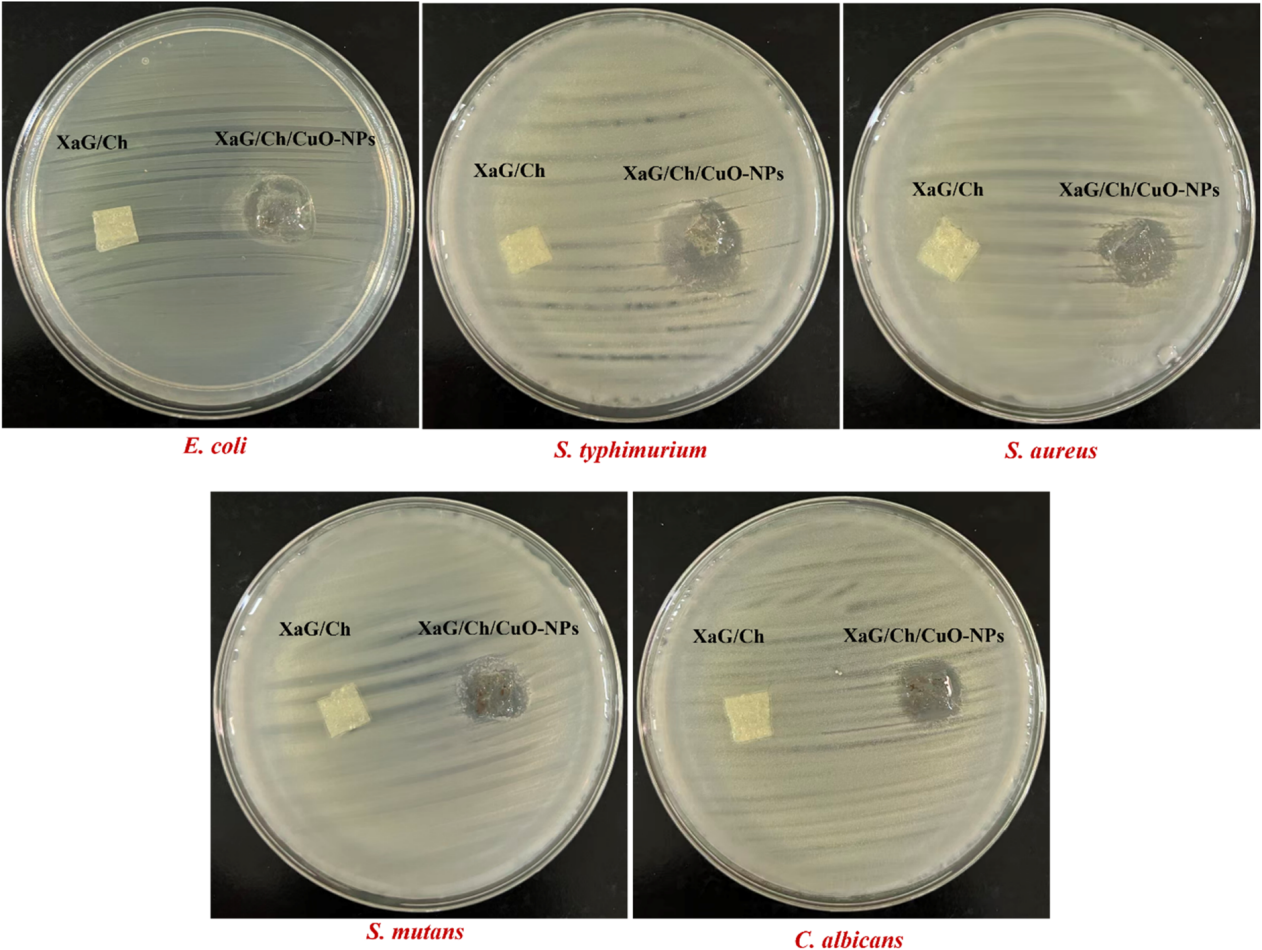
Antimicrobial activity of XaG/Ch and XaG/Ch/CuO-NPs nanocomposite against five microbes using the disc diffusion technique.

The qualitative antimicrobial activity of the XaG/Ch and XaG/Ch/CuO-NPs nanocomposites was evaluated against five pathogenic microorganisms. As shown in Fig. 11, the XaG/Ch nanocomposite exhibited no detectable antimicrobial effect against any of the tested pathogens. This confirms that both XaG and Ch, in their combined form, do not impart inhibitory activity under the tested conditions. These findings are consistent with other reports. Almuthaybiri *et al.* demonstrated that XaG alone lacks antibacterial efficacy due to its anionic nature, which prevents effective interaction with the negatively charged bacterial cell membranes, thereby limiting its ability to induce membrane disruption or growth inhibition.^51^ Similarly, Zayed *et al.* reported that Ch-based collagen gels exhibited negligible antimicrobial activity against all tested pathogenic microorganisms.^52^ Based on the obtained results, the XaG/Ch nanocomposite exhibited limited biological activity. To overcome this limitation, CuO-NPs were incorporated into the XaG/Ch matrix, aiming to enhance the antimicrobial performance of the resulting nanocomposite significantly. The antimicrobial assessment of the XaG/Ch/CuO-NPs nanocomposite against multiple pathogens demonstrates its broad antimicrobial effectiveness, with distinct levels of activity among the tested pathogens. The results revealed that the XaG/Ch/CuO-NPs nanocomposite exhibited comparable inhibition zone diameters for *E. coli*, *S. aureus*, and *C. albicans* (15 ± 1.8 mm), with a slightly lower but non-significant zone for *S. typhimurium* (14 ± 1.3 mm). While *S. mutans* showed the smallest inhibition zone (12 ± 1.2 mm). These observations confirm that XaG/Ch/CuO-NPs nanocomposite shows effective antimicrobial performance against bacteria and unicellular fungi. Collectively, these observations suggest that it can serve as an effective multifunctional antimicrobial agent against diverse pathogens.^53^ Several studies demonstrated that the antimicrobial efficiency of fabricated nanocomposites is due to the decoration with CuO-NPs. For example, Tran with his group reported that the fabricated composite based on cellulose/Ch/CuO-NPs and cellulose/keratin/CuO-NPs composites exhibits excellent antimicrobial activity against *S. aureus*, *E. coli*, *Streptococcus agalactiae*, *Pseudomonas aeruginosa*, *Stenotrophomonas maltophilia*, and *C*. *albicans*. The antibacterial efficiency was found to be lined with the CuO-NPs decorated on the composites.^54^ Another study reports that the nanocomposite film designated Ch, which capped CuO-NPs to form the Ch/CuO-NPs film, has significant antibacterial activity against both Gram-negative and Gram-positive bacteria.^55^ Another report characterized a Ch/polyvinyl alcohol/CuO-NPs nanocomposite, which demonstrated inhibition in *E. coli* at 20.52 ± 0.85 mm and *Bacillus subtilis* at 19.64±0.87 mm.^56^ The biological evaluation of various CuO-NP-based materials exhibits their promising antimicrobial potential, with activity levels highly dependent on the composite structure. Overall, the data obtained in Table 5 represent that CuO-NP offer a versatile platform for antimicrobial applications, where their efficacy can be tailored through strategic combination with other materials such as polymers, bioactive compounds, or other metal oxides. The antimicrobial activity of CuO-NPs has been widely reported; however, the precise mechanisms underlying their action remain incompletely understood. One proposed mechanism involves the interaction of CuO-NPs with the lipid bilayer of microbial cell membranes, leading to membrane disruption, increased permeability, and leakage of intracellular components. This membrane damage can impair the uptake of essential nutrients and compromise cellular integrity, ultimately inhibiting microbial growth.^57^ Furthermore, the gradual release of Cu^2+^ ions from CuO-NPs can interact with proteins, enzymes, and nucleic acids, causing protein denaturation, enzyme inactivation, disruption of DNA replication, and inhibition of ATP synthesis, all of which adversely affect cellular metabolism and viability.^58^ In addition, Cu^2+^ ions can catalyze the generation of reactive oxygen species (ROS), including hydroxyl radicals, superoxide anions, and hydrogen peroxide. The excessive accumulation of ROS induces oxidative stress, resulting in lipid peroxidation, protein oxidation, DNA damage, and ultimately cell death. Therefore, the antimicrobial efficacy of CuO-NPs is generally attributed to the combined effects of membrane disruption, metal ion release, metabolic interference, and ROS-mediated oxidative damage.^59^ In summary, the XaG/Ch/CuO-NPs nanocomposite demonstrated clear antimicrobial potential, evidenced by the formation of distinct inhibition zones in the disc diffusion assay.

**Table 5:** Overview of the antimicrobial profile of the XaG/Ch/CuO-NPs nanocomposite compared with other reported composites decorated with CuO-NP

No.	Composite materials	CuO-NPs fabrication	Microbial strain	Reference
1	XaG/Ch/CuO-NPs	*In situ* using ascorbic acid	*E. coli*, *S. typhimurium, S. aureus*, *S. mutans,* and *C. albicans*	The current study
2	Graphene oxide/TiO_2_/CuO-NPs	*Ex situ* using *Trichoderma virens* filtrate	*S. aureus*, *E. coli*, *S. typhimurium*, *Clavibacter michiganensis*, and *Xanthomonas citri pv*	60
3	Bacterial cellulose/Ch/polyvinyl alcohol/CuO-NPs	*Ex situ* using pomegranate peel extract	*E. coli*, *K. pneumoniae*, *S. typhimurium*, *P. fluorescens*, *A. hydrophila*, *B. subtilis*, *S. aureus*, *S. mutans*, and *C. albicans*	20
4	Aspartic acid/ciprofloxacin/polyethylene glycol/CuO-NPs	*Ex situ* using *Moringa oleifera* roots	*S. typhi*, *P. aeruginosa*, *E. coli*, and *K. pneumoniae*	61
5	Curcumin/CuO-NPs	*Ex situ* using *Penicillium chrysogenum filtrate*	*P. aeruginosa*	62
6	Carbon-NPs/CuO-NPs	*In situ* using the pulsed laser ablation technique	*S. aureus* and *E. coli*	63
7	Nanochitosan/CuO-NPs	*Ex situ* using *Aspergillus* sp filtrate	*E. coli*	57

## 4. Conclusion

In the current study, XaG/Ch/CuO-NPs nanocomposite was synthesized and demonstrated as a promising antimicrobial biosorbent for the removal of organic dyes from aqueous solutions. FT-IR results confirm the successful incorporation of CuO-NPs into the XaG/Ch hydrogel through coordination with hydroxyl, amino, and carboxylate groups to form a stable hydrogel. SEM and EDX confirm the incorporation and uniform dispersion of CuO-NPs within the XaG/Ch hydrogel matrix, resulting in a roughened, reinforced morphology and verifying the presence of elemental copper, indicative of effective *in situ* nanoparticle formation. The incorporation of Cu nanoparticles significantly enhances the thermal stability of the XaG/Ch/CuO-NPs nanocomposite by delaying polymer degradation, promoting char formation, and increasing the residual mass due to thermally stable Cu/CuO species. The adsorption studies demonstrate that the XaG/Ch/CuO-NPs nanocomposite is highly efficient and pH-responsive for both cationic (MB) and anionic (MO) dyes, with adsorption predominantly governed by chemisorption following pseudo-second-order kinetics, Langmuir monolayer behavior, and a spontaneous, endothermic thermodynamic profile.

## Author contributions

A. K. S.: conceptualization, formal analysis, methodology, writing – original draft, review & editing, data curation, visualization, validation. H. A.: formal analysis, writing – original draft, review & editing. J. S. A.: formal analysis, methodology, writing – original draft, review & editing, data curation. F. F. H.: conceptualization, formal analysis, methodology, writing – original draft, review & editing, data curation, visualization, validation.

## Conflicts of interest

There are no conflicts to declare.

## Data Availability

All data generated or analyzed during this study are included in this published article. During the preparation of this work, the authors used ChatGPT in order to optimize English spelling and help in preparation of the graphical abstract. After using this tool/service, the author(s) reviewed and edited the content as needed and take(s) full responsibility for the content of the published article.
